# Comparison of GenesWell BCT Score With Oncotype DX Recurrence Score for Risk Classification in Asian Women With Hormone Receptor-Positive, HER2-Negative Early Breast Cancer

**DOI:** 10.3389/fonc.2019.00667

**Published:** 2019-07-24

**Authors:** Mi Jeong Kwon, Jeong Eon Lee, Joon Jeong, Sang Uk Woo, Jinil Han, Byeong-il Kang, Jee-Eun Kim, Youngho Moon, Sae Byul Lee, Seonghoon Lee, Yoon-La Choi, Youngmi Kwon, Kyoung Song, Gyungyub Gong, Young Kee Shin

**Affiliations:** ^1^Department of Pharmacy, College of Pharmacy, Kyungpook National University, Daegu, South Korea; ^2^Research Institute of Pharmaceutical Sciences, Kyungpook National University, Daegu, South Korea; ^3^Department of Health Sciences and Technology, Samsung Advanced Institute for Health Sciences and Technology (SAIHST), Sungkyunkwan University, Seoul, South Korea; ^4^Department of Surgery, Samsung Medical Center, Sungkyunkwan University School of Medicine, Seoul, South Korea; ^5^Department of Surgery, Gangnam Severance Hospital, Yonsei University College of Medicine, Seoul, South Korea; ^6^Department of Surgery, Korea University Guro Hospital, Seoul, South Korea; ^7^Gencurix, Inc., Seoul, South Korea; ^8^Division of Breast Surgery, Department of Surgery, Asan Medical Center, University of Ulsan College of Medicine, Seoul, South Korea; ^9^Laboratory of Cancer Genomics and Molecular Pathology, Samsung Medical Center, Sungkyunkwan University School of Medicine, Seoul, South Korea; ^10^Department of Pathology and Translational Genomics, Samsung Medical Center, Sungkyunkwan University School of Medicine, Seoul, South Korea; ^11^Center for Breast Cancer, National Cancer Center, Goyang-si, South Korea; ^12^LOGONE Bio Convergence Research Foundation, Seoul, South Korea; ^13^Department of Pathology, Asan Medical Center, University of Ulsan College of Medicine, Seoul, South Korea; ^14^Laboratory of Molecular Pathology and Cancer Genomics, College of Pharmacy, Seoul National University, Seoul, South Korea; ^15^Department of Molecular Medicine and Biopharmaceutical Sciences, Graduate School of Convergence Science and Technology, Seoul National University, Seoul, South Korea

**Keywords:** GenesWell BCT score, oncotype DX recurrence score, concordance, early breast cancer, risk classification, Asian population

## Abstract

**Introduction:** The GenesWell Breast Cancer Test (BCT) is a recently developed multigene assay that predicts the risk of distant recurrence in patients with early breast cancer. Here, we analyzed the concordance of the BCT score with the Oncotype DX recurrence score (RS) for risk stratification in Asian patients with pN0-N1, hormone receptor-positive, human epidermal growth factor receptor 2 (HER2)-negative breast cancer.

**Methods:** Formalin-fixed, paraffin-embedded breast cancer tissues previously analyzed using the Oncotype DX test were assessed using the GenesWell BCT test. The risk stratification by the two tests was then compared.

**Results:** A total of 771 patients from five institutions in Korea were analyzed. According to the BCT score, 527 (68.4%) patients were classified as low risk, and 244 (31.6%) as high risk. Meanwhile, 134 (17.4%), 516 (66.9%), and 121 (15.7%) patients were categorized into the low-, intermediate-, and high-risk groups, respectively, according to the RS ranges used in the TAILORx. The BCT high-risk group was significantly associated with advanced lymph node status, whereas no association between RS risk groups and nodal status was observed. The concordance between the two risk stratification methods in the overall population was 71.9% when the RS low-risk, and intermediate-risk groups were combined into one group. However, poor concordance was observed in patients aged ≤50 years and in those with lymph node-positive breast cancer.

**Conclusions:** The concordance between the BCT score and RS was low in women aged ≤50 years or with lymph node-positive breast cancer. Further studies are necessary to identify more accurate tests for predicting prognosis and chemotherapy benefit in this subpopulation.

## Introduction

Several multigene expression prognostic assays have been developed to overcome the limitations of clinical variables such as tumor size and nodal status for predicting prognosis in breast cancer (1). These assays are used to predict the risk of recurrence or distant metastasis after surgery and adjuvant hormone therapy in hormone receptor-positive early breast cancer to help treatment decisions regarding chemotherapy. MammaPrint (2) and Oncotype DX (3) are the first generation molecular prognostic assays; additional assays such as Prosigna (4–6) and EndoPredict (7) were developed later.

Oncotype DX (Genomic Health, Redwood City, CA, USA) is the most widely used multigene assay (3); it uses quantitative reverse transcription-polymerase chain reaction (qRT-PCR) to measure the expression of 21 genes in formalin-fixed, paraffin-embedded (FFPE) tissues. The Oncotype DX recurrence score (RS) also predicts the benefit of adding chemotherapy to hormone therapy in estrogen receptor (ER)-positive breast cancer (8, 9). Moreover, RS results are currently included in clinical guidelines for treatment decisions in early breast cancer (10–12). The American Joint Committee on Cancer eighth edition cancer staging system was recently revised to include this score for prognosis in breast cancer (13).

However, recent studies showed that other prognostic scores such as PAM50-based Prosigna risk of recurrence (ROR) score (6) and EPclin by EndoPredict (14) are more accurate than Oncotype DX RS for predicting the risk of distant recurrence in endocrine-treated postmenopausal patients with ER-positive breast cancer. Comparison of the prognostic value of six multigene signatures, including Clinical Treatment Score, four immunohistochemical markers (IHC4), RS, ROR, Breast Cancer Index (BCI), and EPclin in 774 postmenopausal women with ER-positive, human epidermal growth factor receptor 2 (HER2)-negative breast cancer also demonstrated that ROR, BCI, and EPclin are more prognostic for overall and late distant recurrence than RS in patients with lymph node-negative breast cancer (15). However, studies comparing Oncotype DX and other assays were performed in Western populations, and the results in Asian patients with breast cancer remain unclear.

Asian breast cancer differs from Western breast cancer in terms of age-specific incidence rates (16–18). Approximately half of breast cancer patients (peak age: 45–50 years) are premenopausal in Asian countries, whereas 15–30% of Western breast cancer (peak age: 55–60 years) are premenopausal (19–21). In addition, distinct biological features of Asian breast cancer include higher prevalence of luminal B subtype, more frequent *TP53* mutation, and more active immune microenvironment, suggesting the needs for inclusion of more Asian women in clinical trials to unravel the ethnic difference of breast cancer (21, 22). However, most genomic algorithms for use in breast cancer tests are based on postmenopausal women in Western countries, which raises concerns regarding their prognostic or predictive value in Asian, or young breast cancer patients. Notably, recent data from the Trial Assigning Individualized Options for Treatment (TAILORx) (23) showed that there is no chemotherapy benefit in patients aged >50 years with hormone receptor-positive, HER2-negative, lymph node-negative breast cancer with a RS of 11–25, while those aged ≤50 years with a RS of 16–25 may benefit from chemotherapy. The trial results suggested that the predictive value of the RS for chemotherapy benefit or “number needed to treat (NNT)” can be different in Asian breast cancer patients, as this population includes a greater number of patients aged ≤50 years. The absolute risk reduction (ARR) and NNT for a RS of 21–25 was 6.5 and 15.4, while it was 1.6 and 62.5 for a RS of 16–20 (23), respectively. Meanwhile, the ARR and NNT for a RS ≥26 was 25.0 and 4.0, respectively (24). A recent study showed that tailored therapy based on Oncotype DX results could result in a net cost increase in initial care of American breast cancer if women aged ≤50 years with tumors with RS of 16–25 all chose to receive chemotherapy (25).

The GenesWell Breast Cancer Test (BCT) (Gencurix, Inc., Seoul, Korea) is a molecular prognostic assay that predicts the risk of 10–year distant metastasis in patients with pathologic N0 or N1 status (pN0-N1), hormone receptor-positive, HER2-negative breast cancer (26). This test is a qRT-PCR-based assay that measures the relative expression of six prognostic genes and two clinical variables using FFPE tumor tissues similar to the Oncotype DX. The ability of this assay to predict the chemotherapy benefit was also recently demonstrated in Asian breast cancer patients (27). Here, we aimed to assess the agreement in risk classification between the BCT score and the RS in a large sample of Asian breast cancer patients from multiple institutions.

## Materials and Methods

### Patients and Tissue Samples

FFPE tumor blocks were obtained from patients meeting the following criteria: with hormone receptor-positive early breast cancer, underwent curative resection of the primary tumor at any of the five institutions (Samsung Medical Center, Asan Medical Center, Korea University Guro Hospital, Gangnam Severance Hospital in Seoul, and National Cancer Institute in Gyeonggi-do) in Korea between 2010 and 2017, and with a reportable RS. FFPE tumor tissues not eligible for the GenesWell BCT test or cases without sufficient tumor or clinical information were excluded. Hormone receptors (ER or progesterone receptor [PR]) and HER2 status were determined at local laboratories. The staining of ER or PR by immunohistochemistry (IHC) was scored using the semi-quantitative Allred score (AS) with a maximum score of 8, and AS >2 was considered as positive as described previously (28, 29). HER2 status was measured using the IHC, fluorescence *in situ* hybridization (FISH), or silver-enhanced *in situ* hybridization (SISH). According to the American Society of Clinical Oncology/College of American Pathologists guidelines, HER2 positivity was defined as an intensity of 3+ by IHC or as gene amplification ratio of ≥2.0 or average HER2 copy number ≥6 by FISH or SISH (30).

### Oncotype DX and BCT Tests

Samples were delivered to Genomic Health for Oncotype DX testing prior to the study. Tissue samples were prepared following the pathology guidelines of Oncotype DX. The RS results were determined by Genomic Health, as previously described (3).

Samples previously analyzed using the Oncotype DX test were used for the GenesWell BCT test. RNA was extracted from FFPE tissues, and samples containing sufficient residual RNA were subjected to qRT-PCR as previously described (26). The BCT score was calculated using two clinical variables (tumor size and nodal status) in combination with the relative expression of the six prognostic genes (*UBE2C, TOP2A, RRM2, FOXM1, MKI67*, and *BTN3A2*) (26). The expression of *ESR1, PGR*, and *ERBB2* was also quantified relative to the three reference genes (*CTBP1, CUL1*, and *UBQLN1*).

### Categorization of Risk Groups

Patients were categorized into BCT high-risk and low-risk groups according to the BCT scoring criteria reported previously (26). Briefly, patients with a BCT score <4 were classified as low risk, and those with a BCT score ≥4 were classified as high risk. For the Oncotype DX, two different RS ranges were used to classify patients. First, patients were grouped into low-risk (RS <18), intermediate-risk (RS 18–30), and high-risk (RS ≥31) groups using the originally validated cut-off (called clinical cut-off) (3). Second, patients were classified according to the RS ranges used in the TAILORx (called TAILORx cut-off) as low-risk (RS <11), intermediate-risk (RS 11–25), and high-risk (RS ≥26) groups (24, 31). Clinical risk was determined using the modified version of Adjuvant! Online as reported in the Microarray in Node-Negative Disease May Avoid Chemotherapy (MINDACT) trial as previously described (27).

### Statistical Analysis

The association between clinicopathological parameters and the BCT score or the RS was analyzed using the Chi-square test. Chi-square test was also used to compare the distribution of each score between the subgroups. The Jonckheere-Terpstra test was used to determine trends in the association between gene expression and risk scores (32, 33). Differences were considered statistically significant at *P* < 0.05. All statistical analyses were performed using R 3.2.0 (http://r-project.org).

## Results

### Patient Characteristics

The GenesWell BCT test was used to analyze 795 FFPE tissue samples from patients with pN0-N1, hormone receptor-positive, HER2-negative breast cancer with available RS results, and the BCT score was calculated for 771 patients. Sample availability is described in Supplementary Figure 1. The clinical characteristics of the patients included in the study are summarized in Table 1. All patients were Asians. The median age was 47 years (range, 23–79 years). A total of 66.7% and 33.3% of the patients were aged ≤50 years and >50 years, respectively. Most of the tumors were ductal carcinoma (85.1%), pN0 (80.3%), histologic grade 2 or 3 (82.2%), and nuclear grade 2 or 3 (91.8%).

**Table 1:** Clinical characteristics of the risk groups according to the BCT score.

			**BCT score**				
**Characteristics**	**All**		**Low risk (<4)**		**High risk (≥4)**		***P*-value**
*n*, %	771		527	68.4%	244	31.6%	–
Age (years)							0.940
≤ 40	135	17.5%	90	66.7%	45	33.3%	
40–50	379	49.2%	260	68.6%	119	31.4%	
50–60	175	22.7%	122	69.7%	53	30.3%	
>60	82	10.6%	55	67.1%	27	32.9%	
ER							-
Positive	771	100.0%	527	68.4%	244	31.6%	
PR							0.470
Negative	78	10.1%	50	64.1%	28	35.9%	
Positive	693	89.9%	477	68.8%	216	31.2%	
Tumor size (cm)							**<0.001**
≤ 2.0	504	65.4%	414	82.1%	90	17.9%	
>2.0	267	34.6%	113	42.3%	154	57.7%	
pN							**<0.001**
0	619	80.3%	457	73.8%	162	26.2%	
1	152	19.7%	70	46.1%	82	53.9%	
Histologic grade							**<0.001**
1	137	17.8%	120	87.6%	17	12.4%	
2	542	70.3%	364	67.2%	178	32.8%	
3	92	11.9%	43	46.7%	49	53.3%	
Nuclear grade							**<0.001**
1	63	8.2%	55	87.3%	8	12.7%	
2	589	76.4%	406	68.9%	183	31.1%	
3	119	15.4%	66	55.5%	53	44.5%	
Histology							0.606
Ductal	656	85.1%	454	69.2%	202	30.8%	
Lobular	67	8.7%	44	65.7%	23	34.3%	
Mucinous	18	2.3%	10	55.6%	8	44.4%	
Others^*^	27	3.5%	18	66.7%	9	33.3%	
Unknown	3	0.4%	1	33.3%	2	66.7%	

* *Cribriform, ductal carcinoma with mucinous, tubular, mixed ductal and lobular, papillary, micropapillary, and metaplastic*.

*BCT, breast cancer test; ER, estrogen receptor; pN, pathologic nodal status; PR, progesterone receptor*.

*ER and PR status was assessed by immunohistochemistry. P < 0.05 are marked in bold*.

### BCT Score-Based Risk Classification

**Figure 1:**
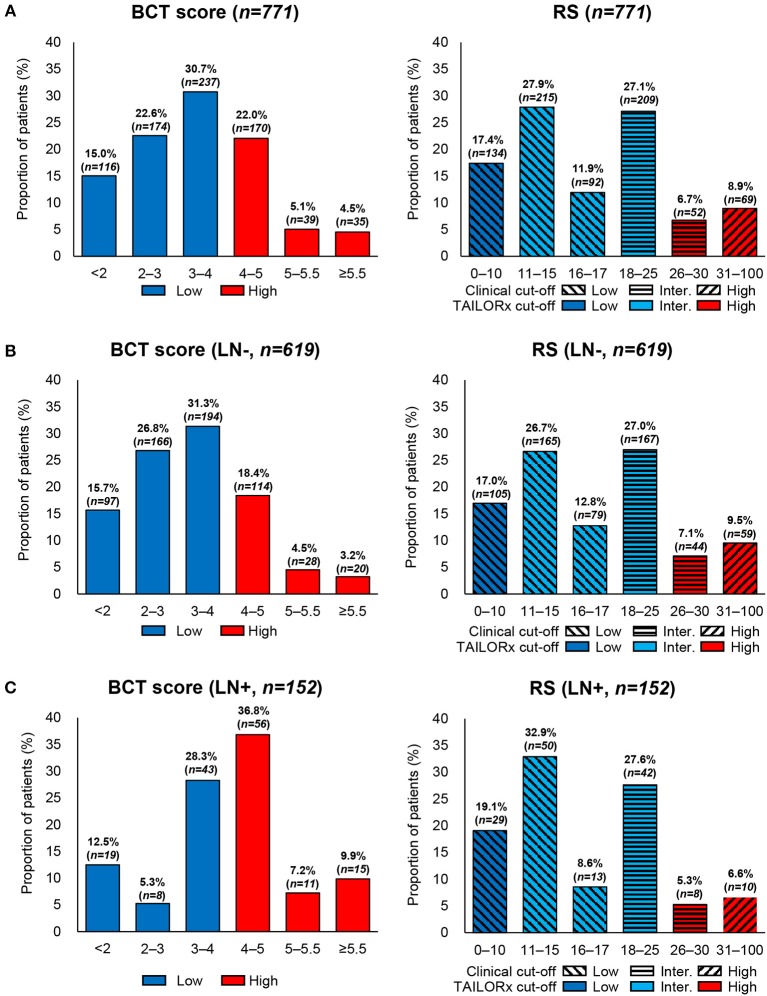
Distribution of the BCT score and Oncotype DX RS by nodal status. Proportion of patients within each risk score range or risk group in **(A)** all patients (*n* = 771), **(B)** lymph node-negative (LN-) patients (*n* = 619), and **(C)** lymph node-positive (LN+) patients (*n* = 152).

**Figure 2:**
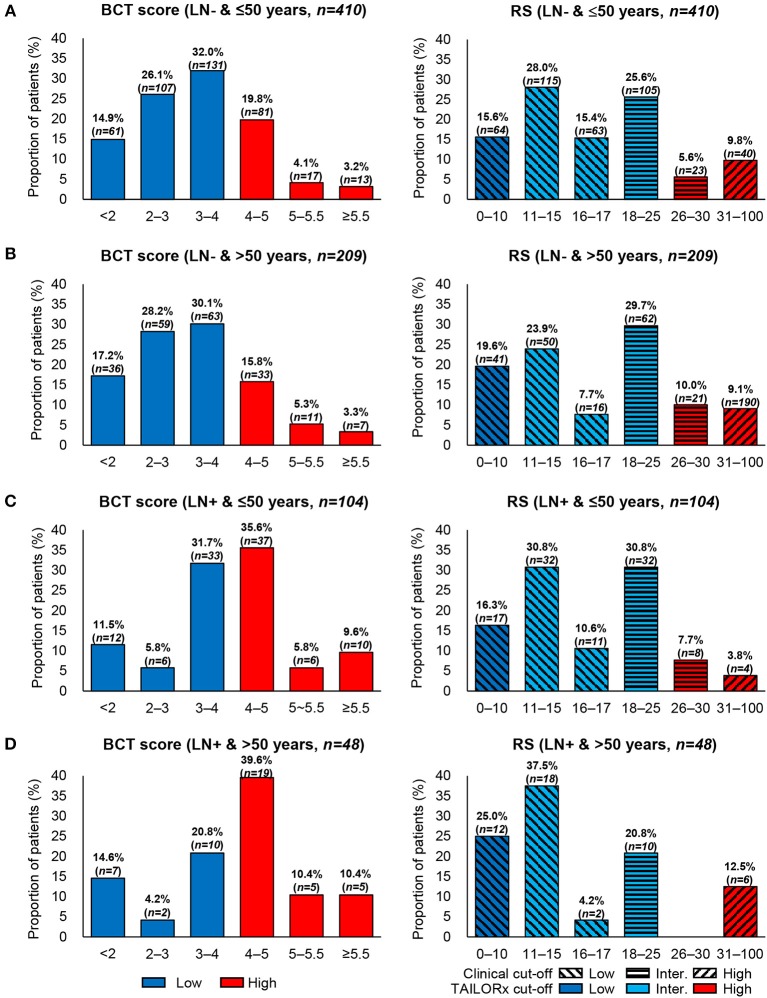
Distribution of the BCT score and Oncotype DX RS by age within each nodal subgroup. Proportion of patients within each risk score range according to age and nodal status. **(A)** Patients aged ≤50 years with lymph node-negative (LN-) breast cancer (*n* = 410). **(B)** Patients aged >50 years with lymph node-negative (LN-) breast cancer (*n* = 209). **(C)** Patients aged ≤50 years with lymph node-positive (LN+) breast cancer (*n* = 104). **(D)** Patients aged >50 years with lymph node-positive (LN+) breast cancer (*n* = 48).

Regarding BCT score distribution, the most common was 3–4 (30.7%), followed by 4–5.5 (27.1%) and 2–3 (22.6%) (Figure 1A). The BCT score distribution differed significantly between lymph node-negative and node-positive subgroups (Figures 1B,C) (*P* < 0.001). Within each nodal subgroup, the BCT score distribution was similar between patients aged ≤50 years and those aged >50 years (*P* = 0.785 for the lymph node-negative subgroup and *P* = 0.694 for the node-positive subgroup) (Figure 2).

In the classification of patients according to the BCT score, 68.4% (*n* = 527) of patients were included in the BCT low-risk group, whereas 31.6% (*n* = 244) were in the BCT high-risk group (Table 1 and Figure 1A). The proportion of BCT high-risk patients was higher in the node-positive (53.9%) than that in the node-negative subgroup (26.1%) (Figures 1B,C). Patients classified into the BCT high-risk group had significantly larger tumors (*P* < 0.001), more advanced pN status (*P* < 0.001), more advanced histologic grade (*P* < 0.001), and higher nuclear grade (*P* < 0.001) than those in the BCT low-risk group. No significant differences in age, PR status and histological type were observed between the two risk groups (Table 1).

### RS-Based Risk Classification

Patients were re-classified as low risk, intermediate risk, and high risk according to the RS results. The most frequent RS range was 11–15 (27.9%), followed by 18–25 (27.1%) (Figure 1A). The RS distribution was similar between the lymph node-negative and node-positive subgroups (*P* = 0.341) (Figures 1B,C). However, a significant difference in the RS distribution according to age was observed in each nodal subgroup (*P* = 0.020 for the lymph node-negative and *P* = 0.035 for the node-positive subgroup) (Figure 2).

Using the original clinical cut-off, 441 (57.2%), 261 (33.9%), and 69 (8.9%) patients were classified as low risk, intermediate risk, and high risk, respectively (Supplementary Table 1). Meanwhile, based on the RS ranges used in TAILORx, 134 (17.4%), 516 (66.9%), and 121 (15.7%) patients were categorized as low risk, intermediate risk, and high risk, respectively (Supplementary Table 1). Compared with the risk classification using the original clinical cut-off, the TAILORx cut-off categorized more patients as intermediate risk and fewer as low risk.

The proportion of patients classified into the high-risk group according to the RS (8.9% using the clinical cut-off and 15.7% using the TAILORx cut-off) was lower than that of patients classified according to the BCT score (31.6%). In contrast to the BCT high-risk group, the RS high-risk group was not significantly associated with advanced pN status. Negative PR status was significantly correlated with a high RS (*P* < 0.001) (Supplementary Table 1).

### Concordance Between the BCT Score and the RS

The concordance in risk stratification between the BCT score and the RS was analyzed using the RS ranges of TAILORx. The overall concordance between the two risk classifications was 71.9% when the RS low-risk and intermediate-risk groups were combined into one group (non-high-risk group, RS 0–25) (Table 2). Of 527 patients in the BCT low-risk group, 480 (91.9%) were classified as non-high risk according to the RS. Subgroup analysis according to nodal status showed that the concordance between the two scores was different in the lymph node-negative and node-positive subgroups. The overall concordance was higher in the lymph node-negative subgroup (76.6%) than that in the node-positive subgroup (52.6%) (Table 2).

**Table 2:** Concordance in risk stratification between the BCT score and Oncotype DX RS according to nodal status and age.

		**All** **(*****n*** **=** **771)**			**Lymph node-negative** **(*****n*** **=** **619)**			**Lymph node-positive** **(*****n*** **=** **152)**		
		**Oncotype DX RS** **(TAILORx cut-off)**			**Oncotype DX RS** **(TAILORx cut-off)**			**Oncotype DX RS** **(TAILORx cut-off)**		
	***n* (%)**	**Non-high risk (0–25)**	**High risk (≥26)**	**Total**	**Non-high risk (0–25)**	**High risk (≥26)**	**Total**	**Non-high risk (0–25)**	**High risk (≥26)**	**Total**
BCT score	Low risk (<4)	480 (62.3%)	47 (6.1%)	527 (68.4%)	414 (66.9%)	43 (6.9%)	457 (73.8%)	66 (43.4%)	4 (2.6%)	70 (46.1%)
	High risk (≥4)	170 (22.0%)	74 (9.6%)	244 (31.6%)	102 (16.5%)	60 (9.7%)	162 (26.2%)	68 (44.7%)	14 (9.2%)	82 (53.9%)
	Total	650 (84.3%)	121 (15.7%)	771 (100.0%)	516 (83.4%)	103 (16.6%)	619 (100.0%)	134 (88.2%)	18 (11.8%)	152 (100.0%)
**≤50 YEARS**										
		**Oncotype DX RS** **(TAILORx cut-off)**			**Oncotype DX RS** **(TAILORx cut-off)**			**Oncotype DX RS** **(TAILORx cut-off)**		
	***n*** **(%)**	**Non-chemobenefit** **(0–15)**	**Chemobenefit** **(≥16)**	**Total**	**Non-chemobenefit** **(0–15)**	**Chemobenefit** **(≥16)**	**Total**	**Non-chemobenefit** **(0–15)**	**Chemobenefit** **(≥16)**	**Total**
BCT score	Low risk (<4)	168 (32.7%)	182 (35.4%)	350 (68.1%)	143 (34.9%)	156 (38.0%)	299 (72.9%)	25 (24.0%)	26 (25.0%)	51 (49.0%)
	High risk (≥4)	60 (11.7%)	104 (20.2%)	164 (31.9%)	36 (8.8%)	75 (18.3%)	111 (27.1%)	24 (23.1%)	29 (27.9%)	53 (51.0%)
	Total	228 (44.4%)	286 (55.6%)	514 (100.0%)	179 (43.7%)	231 (56.3%)	410 (100.0%)	49 (47.1%)	55 (52.9%)	104 (100.0%)
**>50 YEARS**										
		**Oncotype DX RS** **(TAILORx cut-off)**			**Oncotype DX RS** **(TAILORx cut-off)**			**Oncotype DX RS** **(TAILORx cut-off)**		
	***n*** **(%)**	**Non-chemobenefit** **(0–25)**	**Chemobenefit** **(≥26)**	**Total**	**Non-chemobenefit** **(0–25)**	**Chemobenefit** **(≥26)**	**Total**	**Non-chemobenefit** **(0–25)**	**Chemobenefit** **(≥26)**	**Total**
BCT score	Low risk (<4)	159 (61.9%)	18 (7.0%)	177 (68.9%)	140 (67.0%)	18 (8.6%)	158 (75.6%)	19 (39.6%)	0 (0.0%)	19 (39.6%)
	High risk (≥4)	52 (20.2%)	28 (10.9%)	80 (31.1%)	29 (13.9%)	22 (10.5%)	51 (24.4%)	23 (47.9%)	6 (12.5%)	29 (60.4%)
	Total	211 (82.1%)	46 (17.9%)	257 (100.0%)	169 (80.9%)	40 (19.1%)	209 (100.0%)	42 (87.5%)	6 (12.5%)	48 (100.0%)

*BCT, Breast Cancer Test; RS, recurrence score; TAILORx, Trial Assigning Individualized Options for Treatment*.

We also assessed the concordance between the two scores according to age: ≤50 years and >50 years. Based on recent findings on the benefits of chemotherapy for patients with a RS midrange score (11–25) from TAILORx (23), patients were categorized into chemobenefit and non-chemobenefit groups using different RS ranges for each age subgroup. In patients aged ≤50 years, those with RS 0–15 and RS ≥16 were categorized into non-chemobenefit and chemobenefit groups, respectively, whereas in patients aged >50 years, the RS ranges used for the classification into non-chemobenefit and chemobenefit groups were RS 0–25 and RS ≥26, respectively. The overall concordance was higher in women aged >50 years (72.8%) than in those aged ≤50 years (52.9%) (Table 2). However, in each nodal subgroup, the concordance results differed between patients aged ≤50 years and those aged >50 years. In patients with lymph node-negative breast cancer, the concordance was higher in those aged >50 years (77.5%) than in those ≤50 years (53.2%) (Table 2). By contrast, in the lymph node-positive subgroup, the concordance was similar between patients aged >50 years (52.1%), and ≤50 years (51.9%) (Table 2). The highest concordance between the two scores was observed in patients aged >50 years with lymph node-negative breast cancer.

### Comparison of Clinical Risk by Modified Adjuvant! Online With the BCT Score and the RS

**Figure 3:**
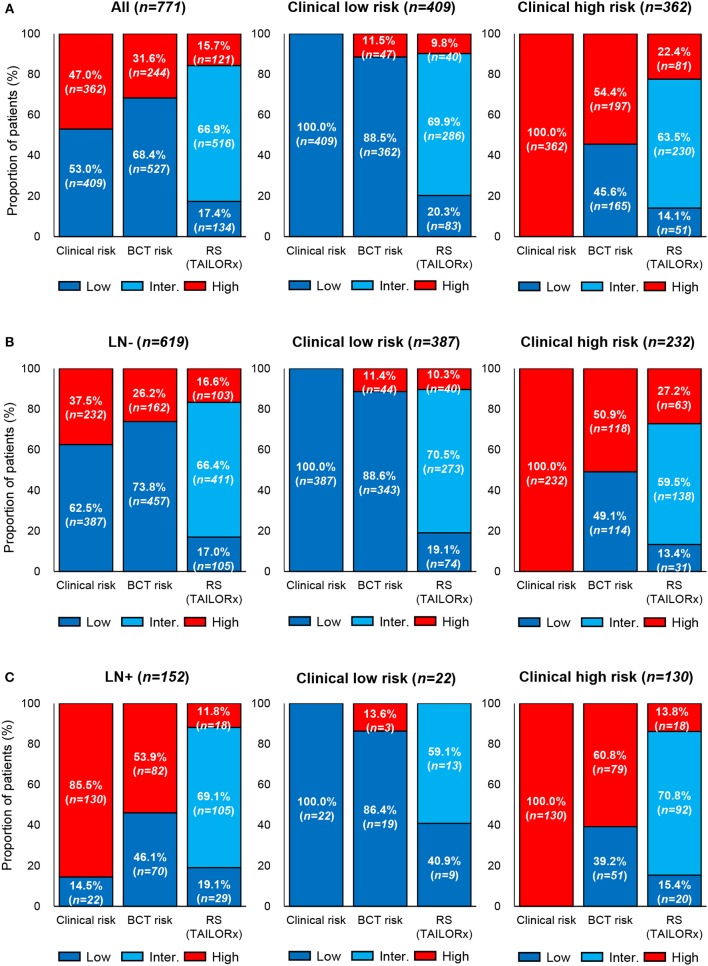
Comparison of clinical risk with the risk classification by the BCT score or Oncotype DX RS. Proportion of patients within each risk group according to clinical risk assessment, BCT score, or RS in **(A)** all patients (*n* = 771), **(B)** lymph node-negative (LN-) patients (*n* = 619), and **(C)** lymph node-positive (LN+) patients (*n* = 152). Clinical risk was determined using the modified Adjuvant! Online, as reported in the MINDACT trial. Risk classification by the RS was based on the recurrence score ranges used in the TAILORx.

The clinical risk of patients was examined using the modified Adjuvant! Online, and the clinical risk classification was compared with that obtained using the BCT score or the RS. Overall, 409 (53.0%), and 362 (47.0%) patients were categorized as clinical low risk and high risk, respectively (Figure 3A). Among patients in the clinical low-risk group, 11.5 and 9.8% were categorized as BCT high risk and RS high risk (≥26), respectively. Among patients in the clinical high-risk group, 45.6% and 77.6% were classified as BCT low risk and RS non-high risk (0–25), respectively. The clinical risk classification according to nodal status was different. The proportion of patients categorized as clinical high risk was higher in the lymph node-positive subgroup (85.5%) than that in the node-negative subgroup (37.5%) (Figures 3B,C). The difference between the clinical risk and the risk stratification using the two tests was greater in the lymph node-positive subgroup than that in the node-negative subgroup.

Of note, a recent secondary analysis of TAILORx trial on the integration of clinical risk to RS showed that the RS ranges predicting chemotherapy benefit are different in young women aged ≤50 years according to clinical risk (34). Clinical low-risk patients with RS 0–20 and RS ≥21 were categorized into non-chemobenefit and chemobenefit groups, whereas in clinical high-risk group, the RS ranges used for the classification into non-chemobenefit and chemobenefit groups were RS 0–15 and RS ≥16, respectively. Based on these findings, we further assessed the concordance between the BCT score and the RS in young patients aged ≤50 years. The overall concordance between the two risk classifications was 66.3% (341/514) and a higher concordance was observed in lymph node-negative subgroup (69.3% [284/410]) than node-positive subgroup (54.8% [57/104]) (Table 3).

**Table 3:** Concordance in risk stratification between the BCT score and Oncotype DX RS in patients aged ≤50 years according to clinical risk.

		**All** **(*****n*** **=** **514)**			**Lymph node-negative** **(*****n*** **=** **410)**			**Lymph node-positive** **(*****n*** **=** **104)**		
		**Oncotype DX RS** **(TAILORx cut-off according to clinical risk)**			**Oncotype DX RS** **(TAILORx cut-off according to clinical risk)**			**Oncotype DX RS** **(TAILORx cut-off according to clinical risk)**		
	***n* (%)**	**Non-chemobenefit** **(0–15 for clinical high risk or 0–20 for clinical low risk)**	**Chemobenefit** **(≥21 for clinical low risk or ≥16 for clinical high risk)**	**Total**	**Non-chemobenefit** **(0–15 for clinical high risk or 0–20 for clinical low risk)**	**Chemobenefit** **(≥21 for clinical low risk or ≥16 for clinical high risk)**	**Total**	**Non-chemobenefit** **(0–15 for clinical high risk or 0–20 for clinical low risk)**	**Chemobenefit** **(≥21 for clinical low risk or ≥16 for clinical high risk)**	**Total**
BCT score	Low risk (<4)	245 (47.7%)	105 (20.4%)	350 (68.1%)	217 (52.9%)	82 (20.0%)	299 (72.9%)	28 (26.9%)	23 (22.1%)	51 (49.0%)
	High risk (≥4)	68 (13.2%)	96 (18.7%)	164 (31.9%)	44 (10.7%)	67 (16.3%)	111 (27.1%)	24 (23.1%)	29 (27.9%)	53 (51.0%)
	Total	313 (60.9%)	201 (39.1%)	514 (100.0%)	261 (63.7%)	149 (36.3%)	410 (100.0%)	52 (50.0%)	52 (50.0%)	104 (100.0%)

*BCT, Breast Cancer Test; RS, recurrence score; TAILORx, Trial Assigning Individualized Options for Treatment*.

*Clinical risk was determined using the modified Adjuvant! Online as reported in the MINDACT trial*.

**Figure 4:**
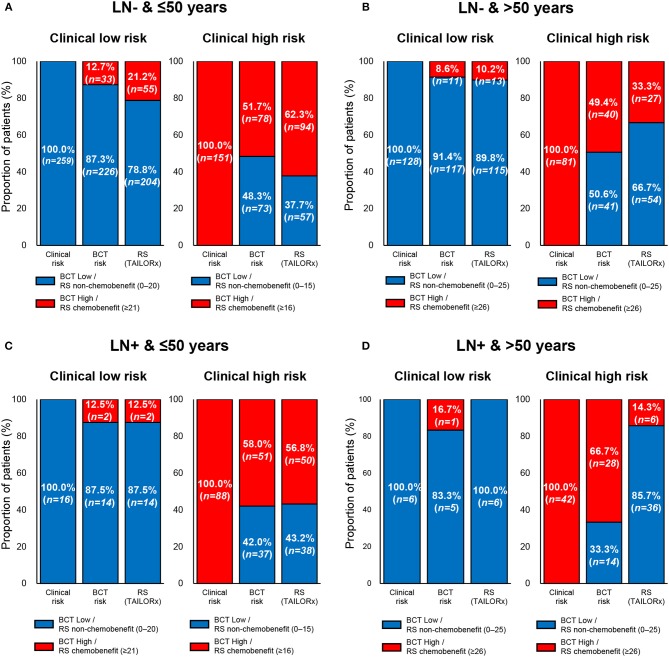
Comparison of clinical risk with the risk classification by the BCT score or Oncotype DX RS by age within each nodal subgroup. Proportion of patients within each risk group according to clinical risk assessment, BCT score, or RS according to age and nodal status. **(A)** Patients aged ≤50 years with lymph node-negative (LN-) breast cancer (*n* = 410). **(B)** Patients aged >50 years with lymph node-negative (LN-) breast cancer (*n* = 209). **(C)** Patients aged ≤50 years with lymph node-positive (LN+) breast cancer (*n* = 104). **(D)** Patients aged >50 years with lymph node-positive (LN+) breast cancer (*n* = 48). Clinical risk was determined using the modified Adjuvant! Online as reported in the MINDACT trial. Patients were divided into non-chemobenefit and chemobenefit groups by different RS ranges according to age group.

Figure 4 shows the discordant results between the clinical risk and the risk classification using the two tests according to age within each nodal subgroup. In both nodal subgroups, the proportion of patients with discordant results between the clinical risk and risk by BCT score (i.e., either clinical low risk and BCT high risk or clinical high risk and BCT low risk) according to age was similar. By contrast, there was a difference in the proportion of patients with discordant results between the clinical risk and RS risk (i.e., either clinical low risk and RS chemobenefit or clinical high risk and RS non-chemobenefit) according to age. The RS categorized a higher proportion of patients into the chemobenefit group among clinical low-risk patients aged ≤50 years (21.2% [55/259] in the lymph node-negative subgroup and 12.5% [2/16] in the node-positive subgroup) than among those aged >50 years (10.2% [13/128] in the lymph node-negative subgroup and 0% [0/6] in the node-positive subgroup). Meanwhile, the proportion of RS non-chemobenefit patients among clinical high-risk patients was higher in women aged >50 years (66.7% [54/81] in the lymph node-negative subgroup and 85.7% [36/42] in the node-positive subgroup) than in those aged ≤50 years (37.7% [57/151] in the lymph node-negative subgroup and 43.2% [38/88] in the node-positive subgroup).

The risk stratification using the two tests in clinical high- or low-risk patients was different in specific subpopulations. In patients aged ≤50 years within the lymph node-negative subgroup (*n* = 259), 21.2% of clinical low-risk patients were categorized into the chemobenefit group according to the RS, whereas 12.7% of patients were categorized as BCT high risk (Figure 4A). Among clinical high-risk patients aged >50 years in the lymph node-positive subgroup (*n* = 42), 33.3 and 85.7% were classified as BCT low risk and non-chemobenefit, respectively, according to the RS (Figure 4D).

The prognostic value of the two scores was difficult to compare because of the short follow-up period. However, seven patients developed distant metastasis after surgery during the follow-up period in the present study. Both the BCT score and the RS categorized four of these patients as high risk (Supplementary Table 2).

### Correlation of ER/PR/HER2 Expression With the BCT Score

**Figure 5:**
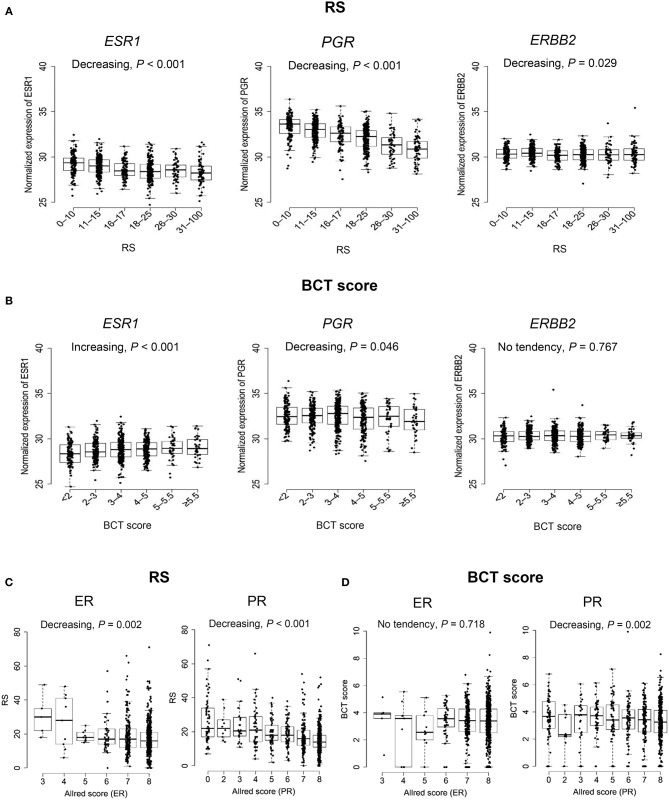
Association between ER, PR, and HER2 expression and the BCT score or Oncotype DX RS. **(A)** Association of *ESR1, PGR*, and *ERBB2* gene expression with the RS and **(B)** the BCT score. **(C)** Correlation of ER and PR expression by Allred score with the RS and **(D)** the BCT score. The *P*-value of the trend was determined using the Jonckheere-Terpstra test (one-sided). The expression of *ESR1, PGR*, and *ERBB2* was measured by qRT-PCR. ER and PR Allred score were determined using immunohistochemistry.

The association of the two scores with the gene expression of *ESR1, PGR*, and *ERBB2* was assessed. Consistent with the RS algorithm including *ESR1* and *PGR* expression, there was a statistically significant trend toward lower *ESR1* and *PGR* expression among patients with a higher RS (Jonckheere-Terpstra test, *P* < 0.001) (Figure 5A). Similarly, *PGR* expression showed a decreasing trend in correlation with the BCT score (*P* = 0.046) (Figure 5B). However, *ESR1* expression increased as the BCT score increased (*P* < 0.001). *ERBB2* expression showed a decreasing trend as the RS increased (*P* = 0.029), whereas no significant association between *ERBB2* expression and the BCT score was observed. We also evaluated the correlation of the two scores with ER and PR expression by IHC. Negative correlation of ER (*P* = 0.002), and PR expression (*P* < 0.001) with the RS was observed (Figure 5C). There was no significant association between ER expression and the BCT score, whereas BCT score showed a negative correlation with PR expression (*P* = 0.002) (Figure 5D).

### Correlation of the RS With BCT Prognostic Genes

The correlation between the expression of six prognostic genes included in the BCT score and the RS was also examined. There was a statistically significant trend toward a higher expression of five proliferation-related genes (*UBE2C, TOP2A, RRM2, FOXM1*, and *MK167*) among patients with a higher RS (Jonckheere-Terpstra test, *P* < 0.001) (Supplementary Figure 2). Although the expression of the immune response-related gene *BTN3A2* was negatively associated with the BCT score, it showed an increasing trend in correlation with the RS (*P* = 0.027).

## Discussion

The present study is the first to compare the BCT score and the RS for the risk classification of Asian patients with pN0-N1, hormone receptor-positive, HER2-negative breast cancer. The study is notable because of the inclusion of a large population of Asian patients from several institutions.

The present results showed a moderate concordance of 71.9% between the two scores for risk stratification using the RS ranges reported in TAILORx. The discrepancy in the risk classification between the BCT score and RS may be attributable to the different gene sets and algorithms used to calculate the score. Moreover, the BCT score algorithm includes clinical factors (tumor size and nodal status), which are not included in the RS. When compared the RS risk group distribution in this study with previous studies, similar distribution was observed. In the present study, 105 (17.0%), 411 (66.4%), and 103 (16.6%) patients were classified as low risk, intermediate risk, and high risk in lymph node-negative subgroup using TAILORx cut-off (Supplementary Table 1), which are similar to results from a TAILORx trial (low risk, 16.7%; intermediate risk, 69.0%, and high risk,14.3%) (23). RS pooled risk group distribution from several studies was: low risk, 52.6%; intermediate risk, 35.9%, and high risk, 11.5%, respectively, when RS risk groups were defined using the original clinical cut-off (35). These results are also similar to our findings.

The results showed that the agreement between the BCT score and the RS differed according to nodal status and age. Better concordance was found in the lymph node-negative subgroup than in the node-positive subgroup and in patients aged >50 years than in those ≤50 years. Accordingly, the highest concordance between the two scores for risk classification was observed in patients aged >50 years with lymph node-negative breast cancer. This was related to the differences in risk assignment by the BCT score or the RS according to nodal status or age. The poor concordance in the lymph node-positive subgroup may be associated with the different risk assignment by the BCT score between the two subgroups. The proportion of patients classified as high risk according to the BCT was higher in lymph node-positive than that in node-negative patients, whereas the RS yielded a similar pattern of risk assignment between the two subgroups. Given that advanced nodal status is a strong unfavorable prognostic factor (36, 37), it is not surprising that the proportion of patients categorized as BCT high risk was higher in the lymph node-positive subgroup than that in the node-negative subgroup. By contrast, the distribution of RS ranges differed between the two age subgroups, whereas the BCT score distribution was similar in each age subgroup. This may explain the large difference in risk stratification by the two risk scores in women aged ≤50 years.

Following the previous TAILORx results, a recent secondary analysis of TAILORx trial further found that clinical risk stratification provided additional prognostic information to hormone receptor-positive, HER2-negative, lymph node-negative breast cancer patients aged ≤50 years with RS 16–25 (34). Importantly, the study showed that there was no benefit from chemotherapy for women aged ≤50 years with RS 16–20 and at clinical low risk, whereas patients with RS 16–25 and at clinical high risk do benefit from chemotherapy. Based on these results, we categorized patients aged ≤50 years into non-chemobenefit and chemobenefit groups using different RS ranges according to clinical risk. Patients with RS 0–20 and RS ≥21 were categorized into non-chemobenefit and chemobenefit groups in clinical low-risk group, whereas in the clinical high-risk group, the RS ranges used for the classification of non-chemobenefit and chemobenefit groups were RS 0−15 and RS ≥16, respectively and we assessed the concordance in risk stratification between the two tests. Similar to the agreement between the two risk classifications not considering clinical risk, the concordance in patients aged ≤50 years was lower than that in patients aged >50 years. The agreement between clinical risk and risk stratification using the two tests varied depending on age. In the subgroup analysis by age in each nodal subgroup, the proportion of patients with discordant results between clinical risk and RS risk was different between patients aged ≤50 years and those >50 years. The risk stratification using the two tests in clinical high- or low-risk patients was different in specific subpopulations including patients aged ≤50 years with lymph node-negative breast cancer and patients aged >50 years with lymph node-positive breast cancer. These results raised a question regarding which risk stratification is more appropriate in these subpopulations. Moreover, these results suggest the need for further studies to identify more accurate risk score for predicting the risk of recurrence or chemotherapy benefit in Asian breast cancer patients aged ≤50 years.

Because the clinical data was based on a short follow-up period, a direct comparison of the prognostic and predictive values of the BCT score with the RS was not possible in this study. Therefore, the results are not sufficient to determine which test is more accurate for predicting the risk of recurrence or chemotherapy benefit in hormone receptor-positive, HER2-negative early breast cancer. However, the BCT high-risk group was significantly associated with larger tumor size and advanced nodal status, whereas the RS showed no significant relationship with nodal status. Moreover, in a recent study that compared the prognostic value of six multigene signatures in postmenopausal patients with ER-positive, HER2-negative breast cancer, combined genomic and clinical models such as ROR and EPclin were more prognostic for late distant recurrence than other molecular signatures in lymph node-positive patients (15). These findings suggest that the BCT score based on combined gene expression and clinical variables, is likely to have a better prognostic value than RS in lymph node-positive patients.

## Conclusions

The present results showed a moderate accordance in risk assignment between the two scores, whereas the concordance was lower in patients aged ≤50 years or those with lymph node-positive disease. Further studies are necessary to directly compare the prognostic and predictive values of the two tests in Asian breast cancer patients aged ≤50 years.

## Data Availability

All datasets generated for this study are included in the manuscript and/or the Supplementary Files.

## Ethics Statement

The study was approved by the review board of five institutions (Samsung Medical Center, Asan Medical Center, Korea University Guro Hospital, Gangnam Severance Hospital in Seoul, and National Cancer Institute in Gyeonggi-do) in Korea and was performed in accordance with the Declaration of Helsinki. Because the study was retrospective in nature, the requirement for informed consent was waived.

## Author Contributions

YKS and GG conceived the study and participated in its design. JEL, JJ, SUW, SBL, SL, Y-LC, and YK were involved in data acquisition. MJK and JH drafted the manuscript. MJK, JEL, JJ, SUW, JH, GG, and YKS analyzed and interpreted the data. JH performed statistical analyses. BK, J-EK, YM, and KS provided administrative, technical, or material support. JEL, JJ, SUW, GG, and YKS participated in critical revision of the manuscript with respect to important intellectual content. YKS supervised the study. All authors read and approved the final manuscript.

### Conflict of Interest Statement

JH, BK, J-EK, and YM are salaried employees of Gencurix. YKS holds a patent application related to the content of this article. The remaining authors declare that the research was conducted in the absence of any commercial or financial relationships that could be construed as a potential conflict of interest.

## References

[1] Gyorffy B Hatzis C Sanft T Hofstatter E Aktas B Pusztai L. Multigene prognostic tests in breast cancer: past, present, future. Breast Cancer Res. (2015) 17:11. 10.1186/s13058-015-0514-2 25848861 PMC4307898

[2] van de Vijver MJ He YD van't Veer LJ Dai H Hart AA Voskuil DW. A gene-expression signature as a predictor of survival in breast cancer. N Engl J Med. (2002) 347:1999–2009. 10.1056/NEJMoa021967 12490681

[3] Paik S Shak S Tang G Kim C Baker J Cronin M. A multigene assay to predict recurrence of tamoxifen-treated, node-negative breast cancer. N Engl J Med. (2004) 351:2817–26. 10.1056/NEJMoa041588 15591335

[4] Parker JS Mullins M Cheang MC Leung S Voduc D Vickery T. Supervisedrisk predictor of breast cancer based on intrinsic subtypes. J Clin Oncol. (2009) 27:1160–7. 10.1200/JCO.2008.18.1370 19204204 PMC2667820

[5] Nielsen TO Parker JS Leung S Voduc D Ebbert M Vickery T. A comparison of PAM50 intrinsic subtyping with immunohistochemistry and clinical prognostic factors in tamoxifen-treated estrogen receptor-positive breast cancer. Clin Cancer Res. (2010) 16:5222–32. 10.1158/1078-0432.CCR-10-1282 20837693 PMC2970720

[6] Dowsett M Sestak I Lopez-Knowles E Sidhu K Dunbier AK Cowens JW. Comparison of PAM50 risk of recurrence score with oncotype DX and IHC4 for predicting risk of distant recurrence after endocrinetherapy. J Clin Oncol. (2013) 31:2783–90. 10.1200/JCO.2012.46.1558 23816962

[7] Filipits M Rudas M Jakesz R Dubsky P Fitzal F Singer CF. A new molecular predictor of distant recurrence in ER-positive, HER2-negative breast cancer adds independent information to conventional clinical risk factors. Clin Cancer Res. (2011) 17:6012–20. 10.1158/1078-0432.CCR-11-0926 21807638

[8] Paik S Tang G Shak S Kim C Baker J Kim W. Gene expression and benefit of chemotherapy in women with node-negative, estrogen receptor-positive breast cancer. J Clin Oncol. (2006) 24:3726–34. 10.1200/JCO.2005.04.7985 16720680

[9] Albain KS Barlow WE Shak S Hortobagyi GN Livingston RB Yeh IT. Prognostic and predictive value of the 21-gene recurrence score assay in postmenopausal women with node-positive, oestrogen-receptor-positive breast cancer on chemotherapy: a retrospective analysis of a randomised trial. Lancet Oncol. (2010) 11:55–65. 10.1016/S1470-2045(09)70314-6 20005174 PMC3058239

[10] Harris LN Ismaila N McShane LM Andre F Collyar DE Gonzalez-Angulo AM. Use of Biomarkers to guide decisions on adjuvant systemic therapy for women with early-stage invasive breast cancer: American society of clinical oncology clinical practice guideline. J Clin Oncol. (2016) 34:1134–50. 10.1200/JCO.2015.65.2289 26858339 PMC4933134

[11] Coates AS Winer EP Goldhirsch A Gelber RD Gnant M Piccart-Gebhart M. Tailoring therapies–improving the management of early breast cancer: St gallen international expert consensus on the primary therapy of early breast cancer 2015. Ann Oncol. (2015) 26:1533–46. 10.1093/annonc/mdv221 25939896 PMC4511219

[12] National Comprehensive Cancer Network. NCCN Clinical Practice Guidelines in Oncology: Breast Cancer Version2.2016. (2017). Available online at: http://www.nccn.org/professionals/physician_gls/pdf/breast.pdf

[13] Giuliano AE Connolly JL Edge SB Mittendorf EA Rugo HS Solin LJ. Breast cancer-major changes in the American joint committee on cancer eighth edition cancer staging manual. CA Cancer J Clin. (2017) 67:290–303. 10.3322/caac.21393 28294295

[14] Buus R Sestak I Kronenwett R Denkert C Dubsky P Krappmann K. Comparison of endopredict and epclin with oncotype DX recurrence score for prediction of risk of distant recurrence after endocrine therapy. J Natl Cancer Inst. (2016) 108:djw149. 10.1093/jnci/djw149 27400969 PMC5241904

[15] Sestak I Buus R Cuzick J Dubsky P Kronenwett R Denkert C. Comparison of the performance of 6 prognostic signatures for estrogen receptor-positive breast cancer: a secondary analysis of a randomized clinical trial. JAMA Oncol. (2018) 4:545–53. 10.1001/jamaoncol.2017.5524 29450494 PMC5885222

[16] Althuis MD Dozier JM Anderson WF Devesa SS Brinton LA. Global trends in breast cancer incidence and mortality 1973-1997. Int J Epidemiol. (2005) 34:405–12. 10.1093/ije/dyh414 15737977

[17] Shin HR Joubert C Boniol M Hery C Ahn SH Won YJ. Recent trends and patterns in breast cancer incidence among Eastern and Southeastern Asian women. Cancer Causes Control. (2010) 21:1777–85. 10.1007/s10552-010-9604-8 20559704

[18] Liu L Zhang J Wu AH Pike MC Deapen D. Invasive breast cancer incidence trends by detailed race/ethnicity and age. Int J Cancer. (2012) 130:395–404. 10.1002/ijc.26004 21351091 PMC3196818

[19] Leong SP Shen ZZ Liu TJ Agarwal G Tajima T Paik NS. Is breast cancer the same disease in Asian and Western countries? World J Surg. (2010) 34:2308–24. 10.1007/s00268-010-0683-1 20607258 PMC2936680

[20] Min SY Kim Z Hur MH Yoon CS Park EH Jung KW. The basic facts of Korean breast cancer in 2013: results of a nationwide survey and breast cancer registry database. J Breast Cancer. (2016) 19:1–7. 10.4048/jbc.2016.19.1.1 27066090 PMC4822102

[21] Kan Z Ding Y Kim J Jung HH Chung W Lal S. Multi-omics profiling of younger Asian breast cancers reveals distinctive molecular signatures. Nat Commun. (2018) 9:1725. 10.1038/s41467-018-04129-4 29713003 PMC5928087

[22] Yap YS Lu YS Tamura K Lee JE Ko EY Park YH. Insights into breast cancer in the east vs. the west: a review. JAMA Oncol. (2019). [Epub ahead of print]. 10.1001/jamaoncol.2019.0620 31095268

[23] Sparano JA Gray RJ Makower DF Pritchard KI Albain KS Hayes DF. Adjuvant chemotherapy guided by a 21-gene expression assay in breast cancer. N Engl J Med. (2018) 379:111–21. 10.1056/NEJMoa1804710 29860917 PMC6172658

[24] Sparano JA Paik S. Development of the 21-gene assay and its application in clinical practice and clinical trials. J Clin Oncol. (2008) 26:721–8. 10.1200/JCO.2007.15.1068 18258979

[25] Mariotto A Jayasekerea J Petkov V Schechter CB Enewold L Helzlsouer KJ. Expected Monetary impact of oncotype DX score-concordant systemic breast cancer therapy based on the TAILORx Trial. J Natl Cancer Inst. (2019) 112. [Epub ahead of print]. 10.1093/jnci/djz068 31165854 PMC7019096

[26] Gong G Kwon MJ Han J Lee HJ Lee SK Lee JE. A new molecular prognostic score for predicting the risk of distant metastasis in patients with HR+/HER2- early breast cancer. Sci Rep. (2017) 7:45554. 10.1038/srep45554 28350001 PMC5368569

[27] Kwon MJ Lee SB Han J Lee JE Lee JW Gong G. BCT score predicts chemotherapy benefit in Asian patients with hormone receptor-positive, HER2-negative, lymph node-negative breastcancer. PLoS ONE. (2018) 13:e0207155. 10.1371/journal.pone.0207155 30462685 PMC6248959

[28] Harvey JM Clark GM Osborne CK Allred DC. Estrogen receptor status byimmunohistochemistry is superior to the ligand-binding assay for predicting response to adjuvant endocrine therapy in breast cancer. J Clin Oncol. (1999) 17:1474–81. 10.1200/JCO.1999.17.5.1474 10334533

[29] Choi YL Oh E Park S Kim Y Park YH Song K. Triple-negative, basal-like, and quintuple-negative breast cancers: better prediction model for survival. BMC Cancer. (2010) 10:507. 10.1186/1471-2407-10-507 20860845 PMC2957395

[30] Wolff AC Hammond ME Hicks DG Dowsett M McShane LM Allison KH. Recommendations forhuman epidermal growth factor receptor 2 testing in breast cancer: American society of clinical oncology/college of American pathologists clinical practice guideline update. Arch Pathol Lab Med. (2014) 138:241–56. 10.5858/arpa.2013-0953-SA 24099077 PMC4086638

[31] Sparano JA Gray RJ Makower DF Pritchard KI Albain KS Hayes DF. Prospective Validation of a 21-gene expression assay in breast cancer. N Engl J Med. (2015) 373:2005–14. 10.1056/NEJMoa1510764 26412349 PMC4701034

[32] Jonckheere R A distribution-free k-sample test against ordered alternatives. Biometrika. (1954) 41:133–45. 10.1093/biomet/41.1-2.133

[33] Terpstra TJ The asymptotic normality and consistency of Kendall's test against trend, when ties are present in one ranking. Indagationes Mathematicae. (1952) 14:327–33. 10.1016/S1385-7258(52)50043-X

[34] Sparano JA Gray RJ Ravdin PM Makower DF Pritchard KI Albain KS. Clinical and genomic risk to guide the use of adjuvant therapy for breast cancer. N Engl J Med. (2019). 380:2395–405. 10.1056/NEJMoa1904819 31157962 PMC6709671

[35] Varga Z Sinn P Fritzsche F von Hochstetter A Noske A Schraml P. Comparison of EndoPredict and Oncotype DX test results in hormone receptor positive invasive breast cancer. PLoS ONE. (2013) 8:e58483. 10.1371/journal.pone.0058483 23505515 PMC3591350

[36] Cianfrocca M Goldstein LJ. Prognostic and predictive factors in early-stage breast cancer. Oncologist. (2004) 9:606–16. 10.1634/theoncologist.9-6-606 15561805

[37] Donegan WL. Tumor-related prognostic factors for breast cancer. CA Cancer J Clin. (1997) 47:28–51. 10.3322/canjclin.47.1.28 8996077

