# Supplementary material for: Comparison of GenesWell BCT Score With Oncotype DX Recurrence Score for Risk Classification in Asian Women With Hormone Receptor-Positive, HER2-Negative Early Breast Cancer
Source: Front Oncol. 2019 Jul 24;9:667. doi: 10.3389/fonc.2019.00667 (PMC6670782; doi:10.3389/fonc.2019.00667)
Supplement: Supplementary file 1 [file Data_Sheet_1.pdf]

Supplementary Figures

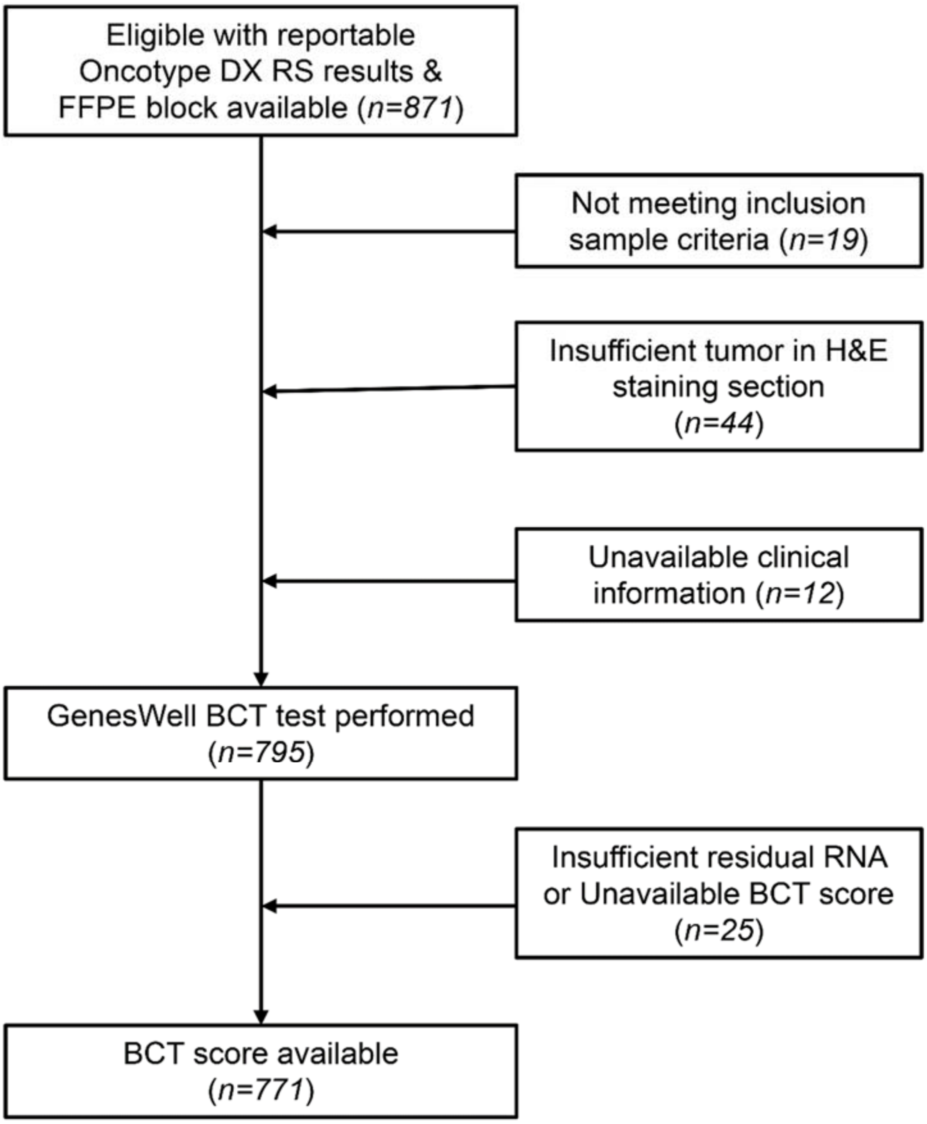

Supplementary Figure 1. Study diagram of the availability of samples for analysis. FFPE, formalin-fixed, paraffin-embedded; H&E, hematoxylin and eosin

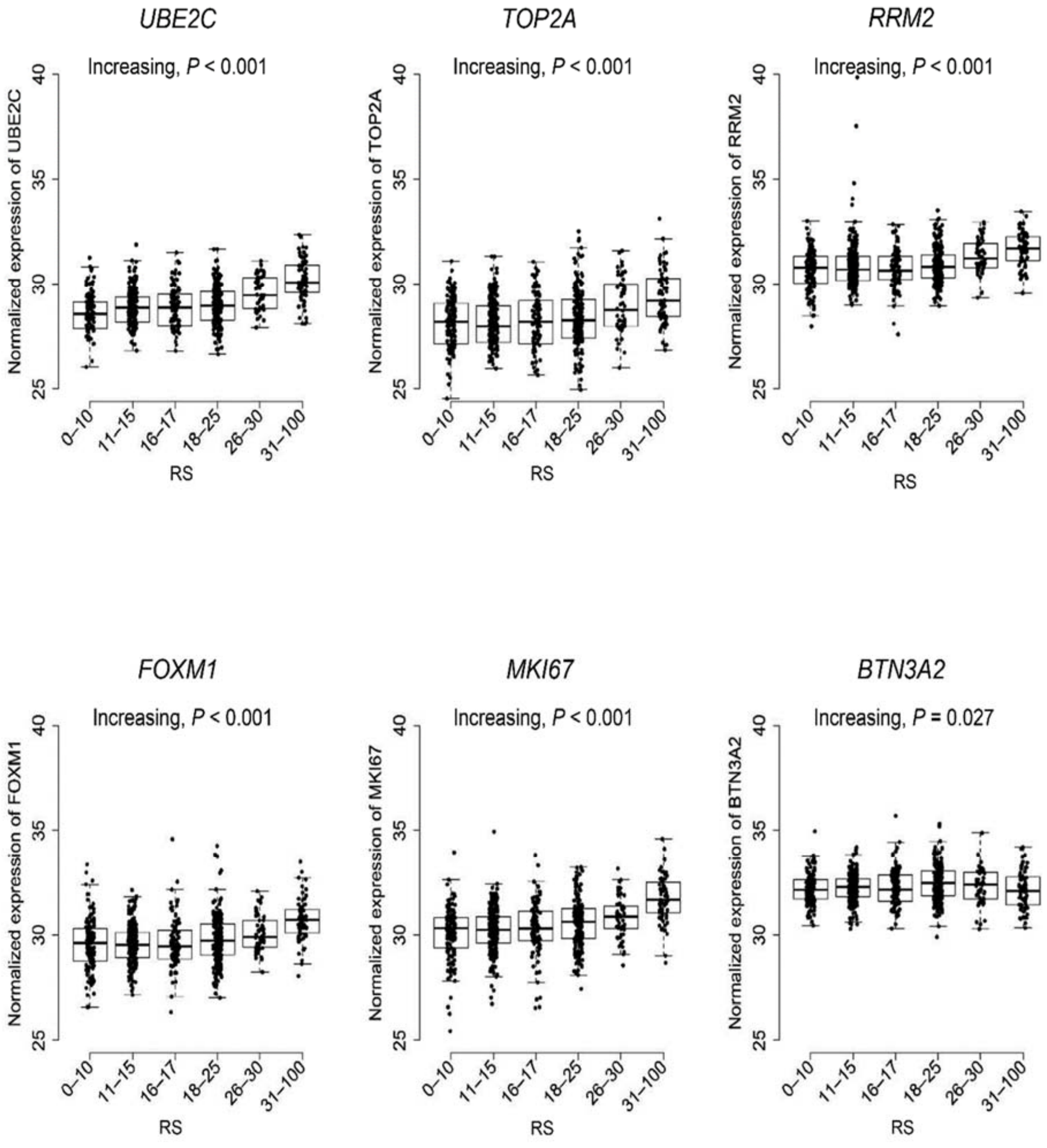

Supplementary Figure 2. Correlation between the expression of BCT prognostic genes and Oncotype DX RS. The expression of five proliferation-related genes (*UBE2C*, *TOP2A*, *RRM2*, *FOXM1*, and *MKI67*) and one immune response-related gene (*BTN3A2*) was determined by qRT-PCR. The Y-axis indicates the normalized gene expression value relative to three reference genes. The *P* value of the trend was determined using the Jonckheere-Terpstra test (one-sided).

Supplementary Tables

Supplementary Table 1. Clinical characteristics of the risk groups according to the Oncotype DX RS

				Oncotype DX RS (Clinical cut-off)							Oncotype DX RS (TAILORx cut-off)						
**Characteristics**		**All**		**Low risk (<18)**		**Intermediate risk (18–30)**		**High risk (≥31)**		***P* value**	**Low risk (<11)**		**Intermediate risk (11–25)**		**High risk (≥26)**		***P* value**
n, %		771		441	57.2%	261	33.9%	69	8.9%	-	134	17.4%	516	66.9%	121	15.7%	-
Age (years)										0.724							0.101
	≤40	135	17.5%	74	54.8%	49	36.3%	12	8.9%		15	11.1%	98	72.6%	22	16.3%	
	40–50	379	49.2%	228	60.2%	119	31.4%	32	8.4%		66	17.4%	260	68.6%	53	14.0%	
	50–60	175	22.7%	94	53.7%	62	35.4%	19	10.9%		33	18.9%	107	61.1%	35	20.0%	
	>60	82	10.6%	45	54.9%	31	37.8%	6	7.3%		20	24.4%	51	62.2%	11	13.4%	
ER										-							-
	Positive	771	100.0%	441	57.2%	261	33.9%	69	8.9%		134	17.4%	516	66.9%	121	15.7%	
PR										**<0.001**							**<0.001**
	Negative	78	10.1%	25	32.1%	29	37.2%	24	30.8%		5	6.4%	39	50.0%	34	43.6%	
	Positive	693	89.9%	416	60.0%	232	33.5%	45	6.5%		129	18.6%	477	68.8%	87	12.6%	
Tumor size (cm)										0.074							**0.006**
	≤2.0	504	65.4%	298	59.1%	169	33.5%	37	7.3%		93	18.5%	347	68.8%	64	12.7%	
	>2.0	267	34.6%	143	53.6%	92	34.5%	32	12.0%		41	15.4%	169	63.3%	57	21.3%	
pN										0.448							0.327
	0	619	80.3%	349	56.4%	211	34.1%	59	9.5%		105	17.0%	411	66.4%	103	16.6%	
	1	152	19.7%	92	60.5%	50	32.9%	10	6.6%		29	19.1%	105	69.1%	18	11.8%	
Histologic grade										**<0.001**							**<0.001**
	1	137	17.8%	100	73.0%	35	25.5%	2	1.5%		36	26.3%	94	68.6%	7	5.1%	
	2	542	70.3%	322	59.4%	185	34.1%	35	6.5%		95	17.5%	378	69.7%	69	12.7%	
	3	92	11.9%	19	20.7%	41	44.6%	32	34.8%		3	3.3%	44	47.8%	45	48.9%	

Nuclear grade										**<0.001**							**<0.001**
1	63	8.2%	44	69.8%	19	30.2%	0	0.0%		14	22.2%	46	73.0%	3	4.8%	
2	589	76.4%	356	60.4%	196	33.3%	37	6.3%		110	18.7%	409	69.4%	70	11.9%	
3	119	15.4%	41	34.5%	46	38.7%	32	26.9%		10	8.4%	61	51.3%	48	40.3%	
Histology									0.051							0.179
Ductal	656	85.1%	368	56.1%	223	34.0%	65	9.9%		113	17.2%	436	66.5%	107	16.3%	
Lobular	67	8.7%	45	67.2%	22	32.8%	0	0.0%		14	20.9%	49	73.1%	4	6.0%	
Mucinous	18	2.3%	12	66.7%	6	33.3%	0	0.0%		2	11.1%	15	83.3%	1	5.6%	
Others*	27	3.5%	16	59.3%	9	33.3%	2	7.4%		5	18.5%	16	59.3%	6	22.2%	
Unknown	3	0.4%	0	0.0%	1	33.3%	2	66.7%		0	0.0%	0	0.0%	3	100.0%	

*Cribriform, ductal carcinoma with mucinous, tubular, mixed ductal and lobular, papillary, micropapillary, and metaplastic
Abbreviations: ER, estrogen receptor; pN, pathologic nodal status; PR, progesterone receptor; RS, recurrence score; TAILORx, Trial Assigning Individualized Options for Treatment
ER and PR status was assessed by immunohistochemistry. *P* values <0.05 are marked in bold.

Supplementary Table 2. Risk classification of patients with distant metastasis according to the BCT score and Oncotype DX RS

Pathologic information							BCT score		Oncotype DX RS		Treatment
**No.**	**Age (years)**	**Tumor size (cm)**	**pN**	**Histologic grade**	**Nuclear grade**	**Clinical risk**	**BCT score**	**Risk group**	**RS**	**Risk group (TAILORx cut-off)**	**Chemotherapy**
1	44	1.5	0	2	2	Low	2.97	Low	6	Low	No
2	57	1.7	0	2	2	Low	4.84	High	32	High	Yes
3	56	1.6	0	2	2	Low	3.51	Low	28	High	Yes
4	52	1.8	0	2	2	Low	2.57	Low	18	Intermediate	No
5	34	1.5	0	3	3	High	4.00	High	49	High	Yes
6	37	1.5	1	2	2	High	4.26	High	21	Intermediate	No
7	43	3.0	1	2	2	High	4.36	High	34	High	Yes

Abbreviations: BCT, Breast Cancer Test; pN, pathologic nodal status; RS, recurrence score; TAILORx, Trial Assigning Individualized Options for Treatment
Clinical risk was determined using the modified version of Adjuvant! Online as reported in the MINDACT trial.
